# Social Determinants and Frailty in High-Need, High-Risk Veterans

**DOI:** 10.1093/geroni/igaa057.578

**Published:** 2020-12-16

**Authors:** Kiranmayee Muralidhar, Willy Marcos Valencia, Fei Tang, Stuti Dang

**Affiliations:** 1 University of Miami, Miami, Florida, United States; 2 Miami VA Healthcare System, Miami, Florida, United States

## Abstract

The VA Geriatrics and Extended Care Data Analysis Center uses national predictive modelling to identify High-Need High-Risk (HNHR) Veterans, to provide targeted services and reduce hospitalization and institutionalization risk. To learn the needs of Miami VA HNHR Veterans, we mailed a needs-assessment survey to 2124 Veterans, of whom 634 responded (29.8% response rate). The average respondent age was 70.5±9.2. Among them, 127(20%) were <65 years old, 326(51.4%) were 65-74, and 179(28.2%) were ≥75; 389(61.4%) White, 225(35.5%) Black/African Americans; 515(81.2%) were Non-Hispanic, 111(17.5%) Hispanic/Latino; 173(27.3%) were high school graduates, 350(55.2%) had at least some college credit, 39(6.2%) had a master’s degree or more and 536(84.5%) were health literate. As per Morley’s FRAIL scale, 266(42%) were frail, 242(38.2%) were pre-frail and 87(13.7%) were robust. Social risk factors possibly associated with frailty were analyzed using ordinal logistic regression. Univariate analysis showed significant association with poor health literacy, having a caregiver, social isolation, transportation trouble, delayed or missed doctors’ appointments due to transportation, a negative perception of aging, likelihood of depression, being homebound, inability to use the internet, lack of technology for video conferencing and lack of email use (p≤0.01). Through multivariate ordinal logistic regression analysis, adjusting for patients’ age and Jen Frailty Index, we found that the same social risk factors other than internet use showed significant association with frailty (p≤0.01). HNHR Veterans have complex social needs with a limited ability to manage their chronic conditions, necessitating interventions that address not only their medical issues but also their access barriers and social support.

